# Evolution of the apomixis transmitting chromosome in *Pennisetum*

**DOI:** 10.1186/1471-2148-11-289

**Published:** 2011-10-05

**Authors:** Yukio Akiyama, Shailendra Goel, Joann A Conner, Wayne W Hanna, Hitomi Yamada-Akiyama, Peggy Ozias-Akins

**Affiliations:** 1 Department of Horticulture, The University of Georgia, 2360 Rainwater Rd., Tifton, GA 31793-5766, USA; 2 Department of Crop and Soil Sciences, The University of Georgia, 2360 Rainwater Rd., Tifton, GA 31793-5766, USA; 3 Graduate School of Agriculture, Iwate University, 3-18-8 Ueda, Morioka, Iwate 020-8550, Japan

## Abstract

**Background:**

Apomixis is an intriguing trait in plants that results in maternal clones through seed reproduction. Apomixis is an elusive, but potentially revolutionary, trait for plant breeding and hybrid seed production. Recent studies arguing that apomicts are not evolutionary dead ends have generated further interest in the evolution of asexual flowering plants.

**Results:**

In the present study, we investigate karyotypic variation in a single chromosome responsible for transmitting apomixis, the Apospory-Specific Genomic Region carrier chromosome, in relation to species phylogeny in the genera *Pennisetum *and *Cenchrus*. A 1 kb region from the 3' end of the *ndh*F gene and a 900 bp region from *trn*L-F were sequenced from 12 apomictic and eight sexual species in the genus *Pennisetum *and allied genus *Cenchrus*. An 800 bp region from the Apospory-Specific Genomic Region also was sequenced from the 12 apomicts. Molecular cytological analysis was conducted in sixteen *Pennisetum *and two *Cenchrus *species. Our results indicate that the Apospory-Specific Genomic Region is shared by all apomictic species while it is absent from all sexual species or cytotypes. Contrary to our previous observations in *Pennisetum squamulatum *and *Cenchrus ciliaris*, retrotransposon sequences of the Opie-2-like family were not closely associated with the Apospory-Specific Genomic Region in all apomictic species, suggesting that they may have been accumulated after the Apospory-Specific Genomic Region originated.

**Conclusions:**

Given that phylogenetic analysis merged *Cenchrus *and newly investigated *Pennisetum *species into a single clade containing a terminal cluster of *Cenchrus *apomicts, the presumed monophyletic origin of *Cenchrus *is supported. The Apospory-Specific Genomic Region likely preceded speciation in *Cenchrus *and its lateral transfer through hybridization and subsequent chromosome repatterning may have contributed to further speciation in the two genera.

## Background

Apomixis is an intriguing trait in plants that allows multiplication of maternal clones through seed reproduction [1]. Besides the potential for apomixis to be a powerful plant breeding tool due to the circumvention of genetic segregation and maintenance of heterosis in hybrid progenies, the trait is also compelling in terms of evolutionary studies. Apomicts have long been regarded as evolutionary dead ends [2] mainly because of their presumed lack of genetic variation in the absence of recombination and intermating, although various studies have shown high levels of chromosomal and morphological variation within agamic complexes [3,4]. More recently, levels of genetic diversity among asexual populations were found to be higher than expected when compared to those in sexually reproducing populations [5-9]. Apomicts can outcross when they produce viable pollen, through the occasional reduced egg, or by fertilization of unreduced eggs, and can thus act as bridges for introgressive hybridization between otherwise reproductively isolated taxa [10-14]. Hybrid lineages can be stabilized by apomixis, allopolyploidy and recombinational speciation [15]. Apomixis does, however, reduce the rate of chromosomal recombination in the female, thereby diminishing the opportunity for unequal crossing over to reduce repetitive element copy number [16], allowing instead an accumulation of transposons in the genome and an increase in genome size, at least in relatively recent lineages [17]. Recombination is further constrained during male meiosis in apomicts in the chromosomal region transmitting the trait to progeny [18,19]. The fundamental importance of recombination and the paradox of sex [20,21] have inspired interest in deciphering the evolution of asexual organisms [14,22].

The *Pennisetum/Cenchrus *branch of the monophyletic bristle clade of grasses [23] contains a major crop species, sexual pearl millet or *Pennisetum glaucum *(L.) R. Br., and at least 17 aposporous species [18]. Relationships have been inferred among some of these species using basic chromosome numbers, ITS (the internal transcribed spacers of ribosomal RNA genes) DNA data [24] and sequences from chloroplast genes such as *ndh*F (F subunit of NADH dehydrogenase) [25], *ndh*F and *trn*L-F [26], *trn*L-F and *rpl*16 [27]. Chemisquy [26] also used a nuclear gene (*knotted*) to study the phylogeny in *Cenchrus*, *Pennisetum *and related genera.

ITS sequences provide limited resolution to estimate genetic similarities of hybrids and their parents due to concerted evolution [28]. Though chloroplast DNA is maternally inherited, and therefore can be criticized for its inability to assess biparental contribution to the genome, it can provide sequences from specific genes or intergenic regions that are phylogenetically informative. The tobacco (*Nicotiana tabacum*) *ndh*F gene is 2223 bp in length and has a nucleotide substitution rate [29] which is, for example, two times greater than that of *rbcL*, a second extensively studied chloroplast gene [30]. More recent studies have also demonstrated that the 3'end of *ndh*F is more variable than the 5' region [31]. For the present study, we chose to sequence two chloroplast gene regions (a 1131-1155 bp fragment from the 3'end of *ndh*F and 811-872 bp region from *trn*L-F) and a 792-799 bp segment from the *ASGR-BBM-like *gene, also located within the p208 BAC used in fluorescence *in situ *hybridization (FISH) analysis. We furthermore report molecular cytogenetic analysis of the genomic region associated with apomixis, the apospory-specific genomic region (ASGR) that was previously identified in *P. squamulatum*, *C. ciliaris *and now in 16 *Pennisetum *and one additional *Cenchrus *species.

The ASGR is conserved between *P. squamulatum *and *C. ciliaris *based on high sequence similarity between putative orthologous genes within this region; syntenic relationships between chromosomal sequences identified by BAC probes; shared cytological features of hemizygosity, the heterochromatic nature of the ASGR, and a region of low copy DNA flanked by high copy sequences [32-37]. Nevertheless, there are distinct structural differences in the ASGR-carrier chromosomes of these two species. These previous observations suggested that a conserved ASGR haplotype may occur in different chromosomal contexts among species. We now compare the extent of conservation and variation in the ASGR and ASGR-carrier chromosome in parallel with a *Pennisetum *and *Cenchrus *species phylogeny constructed with sequence data from chloroplast genes, *ndh*F and *trn*L-F. Variability observed in chromosomal context should enable a more precise delineation of the ASGR.

## Results

### Phylogenetic analysis based on *ndh*F and *trn*L-F sequences

All species (Table 1) generated a 3' *ndh*F sequence of 1134 bp except for *P. hohenackeri *(PS156) and *P. polystachion *(PS19). PS156 had an insertion of 21 bp while PS19 showed a 3 bp deletion. For the *trn*L-F region, size varied from 863 bp to 872 bp except in the case of *P. polystachion*, *P. pedicillatum *and *P. subangustum *which showed a length of 811 bp. *ndh*F and *trnL-F *produced an aligned matrix of 1155 and 901 nucleotide positions respectively thus giving a total aligned matrix of 2056 characters. The matrix had 1913 constant, 76 parsimony uninformative and 67 parsimony informative characters. A partition homogeneity test was done for 100 replicates, although the test was aborted during the 78^th ^replicate due to time constraints (655:46 hr). The test gave a P-value of 0.86 supporting the combination of data sets for analysis.

**Table 1:** Plant materials

Species	Primary ID	Secondary ID	Reported Chromosome No.	Ploidy	Reported MOR
*C. ciliaris*	PS185	'LLANO'	36	4x	A
*C. ciliaris*	PS186	'NUECES'	36	4x	A
*C. setigerus*	PS16	PI266185	36	4x	A
*P. alopecuroides*	PS938	9064-3	18	2x	S
*P. basedowii*	PS2	PI257782	54	6x	S
*P. flaccidum*	PS32	PI271601	18,36,45	2x,4x,5x	S,A
*P. flaccidum*	PS95	TIMOTHY C79I3	18,36,45	2x,4x,5x	S,A
*P. glaucum*	23BE	-	14	2x	S
*P. hohenackeri*	PS156	ICRISAT	18	2x	S
*P. massaicum*	PS680	IBPCR	36	4x	A
*P. massaicum*	PS953	WIPFF 87A11508	36	4x	A
*P. mezianum*	PS9	PI365021	16, 32	2x,4x	S,A
*P. nervosum*	PS187	#7-82	36,72	4x,8x	S
*P. nervosum*	PS38	PI316421	36,72	4x,8x	S
*P. orientale*	PS12	PI315867	18,27,36,45,54	2x-6x	S,A
*P. orientale*	PS13	PI218097	18,27,36,45,54	2x-6x	S,A
*P. pedicillatum*	PS304	HARLAN 682	36,54	4x,6x	A
*P. polystachion*	PS19	PI189347	36,54,63	4x,6x,7x	A
*P. purpureum*	N109	-	28	4x	S
*P. purpureum*	N168	-	28	4x	S
*P. ramosum*	PS29	PI331699	10	2x	S
*P. ramosum*	PS63	DEWET1641	10	2x	S
*P. schweinfurthii*	PS243	PI489685	14	2x	S
*P. setaceum*	PS22	PI300087	27,54	3x,6x	A
*P. setaceum*	PS25	PI364994	27,54	3x,6x	A
*P. squamulatum*	PS158	ICRISAT	54	6x	A
*P. squamulatum*	PS24	PI248534	54	6x	A
*P. subangustum*	PS163	IBADAN#2	36,54	4x,6x	A
*P. villosum*	PS249	TEL AVIV	18,27,36,45,54	2x-6x	S,A
*Setaria viridis*	GI:758770	-	18	2x	S

List of *Cenchrus *and *Pennisetum *species with corresponding identifiers, mode of reproduction and chromosome data. Reported chromosome numbers are from Jauhar [57], Dujardin and Hanna [57] and the Kew C-value database (http://data.kew.org/cvalues/). MOR = Mode of Reproduction; A = apomictic; S = sexual.

A simple heuristic search of the aligned matrix using Phylogenetic Analysis Using Parsimony (PAUP) retained 28 trees. All trees were 166 steps in length and had a consistency index (CI) of 0.875, retention index (RI) of 0.895 and rescaled consistency index (RC) of 0.775. The log likelihood of all the trees ranged from 3974.04078 to 3971.98984. To account for homoplasy generated by gaps, the gap creating regions were ignored (accounting for ~97 characters of aligned matrix). After exclusion, a heuristic search generated 9 trees each showing a length of 152 steps with CI of 0.875, RI of 0.914 and RC of 0.799. The log likelihood for all the trees ranged from 3755.09184 to 3757.65531.

**Figure 1:**
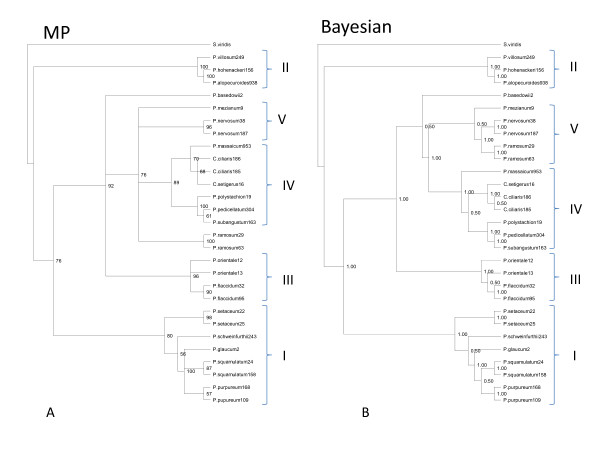
**Maximum Parsimony and Bayesian tree based on *ndh*F and *trn*L-F**. Maximum parsimony (MP) and Bayesian trees based on the *ndh*F+*trn*LF sequence alignments generated in the present study. Numbers at the nodes show bootstrap values obtained.

Phylogenetic trees with similar topologies were generated by Bayesian and maximum parsimony (MP) methods. Overall five groups emerged in the present phylogenetic study (Figure 1A and 1B, Table 2). All major groups showed good bootstrap support except that the group of *P. ramosum*, *P. nervosum *and *P. mezianum *showed low support in the Bayesian-based analysis. These species also showed variation with respect to their position in the two trees (Bayesian and Maximum Parsimony). Subgroups I, II, and V contain apomictic and obligately sexual species whereas subgroups III and IV contain apomictic species with sexual cytotypes or facultative apomixis.

**Table 2:** Species clusters and cytological characteristics

Group No. in MP Tree	Group No in Bayesian Tree	Species Name	Primary ID	MOR	Number of chromo-somes observed	Number of ASGR	25S rDNA on ASGR-carrier chromosome	Opie-2 around ASGR	Opie-2 in genome	Enzyme treatment (min)/Denature (sec)
I	I	*P. glaucum*	23BE	S	14	0	-	-	low	80/70
I	I	*P. purpureum*	N109	S	-	-	-	-	-	-
I	I	*P. purpureum*	N168	S	-	-	-	-	-	-
I	I	*P. schweinfurthii*	PS243	S	14	0	-	-	high	80/90
I	I	*P. setaceum*	PS22	A	-	-	-	-	-	-
I	I	*P. setaceum*	PS25	A	27	1	no	no	mid	80/90
I	I	*P. squamulatum*	PS158	A	56	1	no	high	low	Ref. [50]
I	I	*P. squamulatum*	PS24	A	56	1	no	high	low	120/90
II	II	*P. alopecuroides*	PS938	S	18	0	-	-	low	80/90
II	II	*P. hohenackeri*	PS156	S	-	-	-	-	-	-
II	II	*P. villosum*	PS249	A	45	1	no	no	mid	80/90
III	III	*P. flaccidum*	PS32	A	36	-	-	-	-	-
III	III	*P. flaccidum*	PS95	A	36	1	no	low	low	100/90
III	III	*P. orientale*	PS12	A	54	2	no	low	low	120/45
III	III	*P. orientale*	PS13	A	-	-	-	-	-	-
IV	IV	*C. ciliaris*	B12-9	A	36	1	yes (same arm)	high	high	Ref. [37]
IV	IV	*C. ciliaris*	Higgins	A	36	1	yes (same arm)	high	high	Ref. [37]
IV	IV	*C. ciliaris*	PS185	A	-	-	-	-	-	-
IV	IV	*C. ciliaris*	PS186	A	-	-	-	-	-	-
IV	IV	*C. setigerus*	PS16	A	36	1	yes (same arm)	high	high	80/90
IV	IV	*P. massaicum*	PS680	A	35	1	yes (diff arm)	no	high (on 27/35 chr)	90/50
IV	IV	*P. massaicum*	PS953	A	35	-	-	-	-	-
IV	IV	*P. pedicillatum*	PS304	A	36	1	no	high	high	80/90
IV	IV	*P. polystachion*	PS19	A	54	1	no	mid	mid	105/70
IV	IV	*P. subangustum*	PS163	A	54	1	no	high	high	60/90
V	V	*P. mezianum*	PS9	A	32	1	no	high	low	80/90
V	V	*P. nervosum*	PS187	S	54	0	-	-	high	100/90
V	V	*P. nervosum*	PS38	S	-	-	-	-	-	-
none	V	*P. ramosum*	PS29	S	-	-	-	-	-	-
none	V	*P. ramosum*	PS63	S	10	0	-	-	low	80/50
none	none	*P. basedowii*	PS2	S	54	0	-	-	low	80/50

Phylogenetic (*ndh*F) and cytological (ASGR and Opie-2 retrotransposon) analyses of *Pennisetum *and *Cenchrus *species.

**Figure 2:**
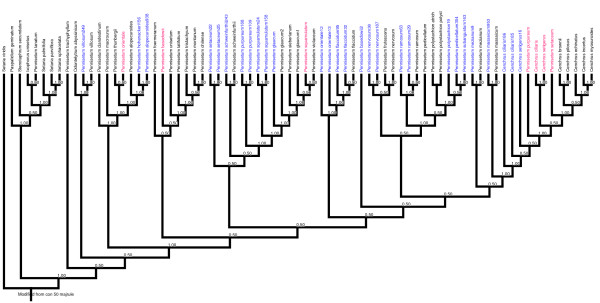
**Comparative Bayesian tree**. Bayesian tree based on *ndh*F+*trn*LF sequence alignments generated from combined analysis of sequence matrix generated in present study and Chemisquy et al. [26].

A recent paper [26] also used *ndh*F and *trn*L-F sequences to understand the relationship among *Pennisetum *and *Cenchrus *species. To compare their analysis with that of the present study, the sequence alignment was downloaded from TreeBase http://purl.org/phylo/treebase/phylows/study/TB2:S10252. The resultant matrix was too large to be analyzed by PAUP, hence it was only analyzed by Mr. Bayes (Figure 2). Seven sequences were removed from the analysis due to substantial amounts of missing data. The taxa used in the present study are shown in blue while those shown in red are from Chemisquy [26] whose grouping does not entirely agree with that generated in the present study.

### Phylogenetic analysis based on sequence from the ASGR region

**Figure 3:**
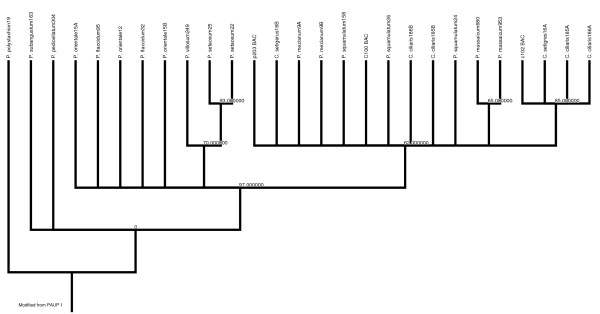
**ASGR-based Maximum Parsimony tree**. Maximum parsimony tree based on the sequence alignments generated from the ASGR in the present study. Numbers at the nodes show bootstrap values obtained.

Eight primer pairs, previously identified as ASGR-linked in F_1 _populations where *P. squamulatum *and *C. ciliaris *were the apomictic parents, were tested on all species used in this study (Additional File 1). Only the primer pair p779/p780 which amplifies a portion of the *ASGR-BBM-like *gene resulted in amplification of all the apomictic species but none of the sexual species. Primers p779/p780 are located in the 4^th ^and 7^th ^exons of *ASGR-BBM-like2 *(EU559277) and amplify a region including 3 introns of 95 bp, 266 bp, and 154 bp. Based on ASGR-linked BAC clone sequencing, *P. squamulatum *and *C. ciliaris *have duplicated *ASGR-BBM-like *genes [38]. The p779/p780 primers amplify both copies, although polymorphism between copies cannot be detected in *P. squamulatum *while polymorphism is detectable in *C. ciliaris*. The present analysis could differentiate two copies of the *ASGR-BBM-like *gene in *C. setigerus*, *P. orientale*, *P. mezianum *and *C. ciliaris*. In *P. orientale*, accession PS12 did not show two copies while PS15 did. Among the two types of sequences obtained in *C. ciliaris *and *C. setigerus*, one showed similarity with *P. squamulatum *while the other sequence grouped with the other copy from *Cenchrus *(Figure 3).

The *ASGR-BBM-like *sequence ranged in size from 792-799 bp. Alignment provided a matrix of 800 bp with 785 constant, 4 uninformative and 11 informative characters. Heuristic search retained only one tree. This tree was only 15 steps in length with trichotomies and a log likelihood score of 1206.32388 (Figure 3).

### Detection of the ASGR-carrier chromosome in apomictic *Pennisetum *species

**Figure 4:**
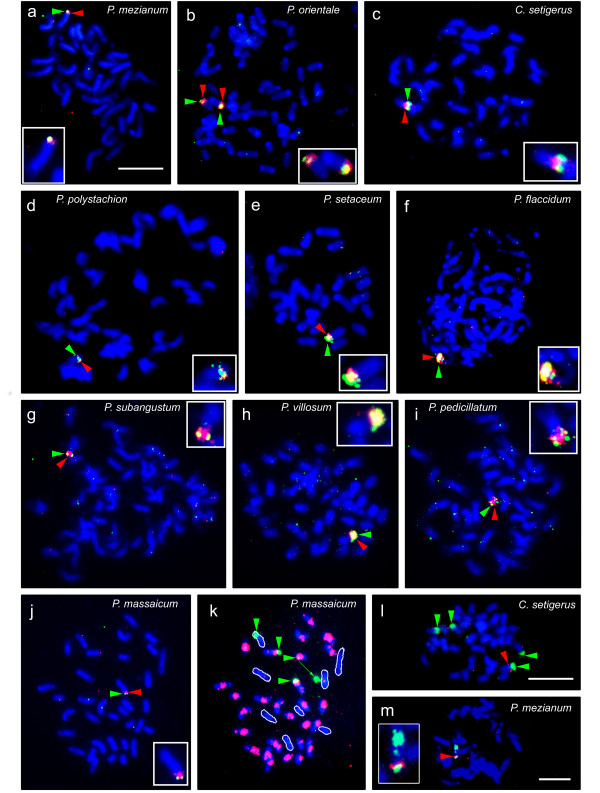
**Physical mapping with ASGR-linked BACs on chromosome spreads from various *Pennisetum *and *Cenchrus *species**. a-j, Color-merged images of FISH signals and inverted DAPI-stained chromosomes; red and green arrows indicate P001 and P208, respectively; insets show enlarged, pseudo-colored ASGR-carrier chromosome. k-m, Images of dual-labeled FISH on DAPI-stained chromosomes. a, m: *P. mezianum *(PS9); b: *P. orientale *(PS12); c: *C. setigerus *(PS16); d: *P. polystachion *(PS19); e: *P. setaceum *(PS25); f: *P. flaccidum *(PS95); g: *P. subangustum *(PS163); h: *P. villosum *(PS249); i: *P. pedicillatum *(PS304); j: *P. massaicum*; k: *P. massaicum *spread in panel j stripped and rehybridized with rDNA and P602 (red); green arrows and signals indicate rDNA; outlined chromosomes did not hybridize with P602. l: *C. setigerus *hybridized with rDNA and P208; green signals are rDNA and red arrow indicates P208 signal. m: *P. mezianum *(PS9) hybridized with P208 (red arrow) and P602 (green signal). Bars correspond to 10 μm.

**Figure 5:**
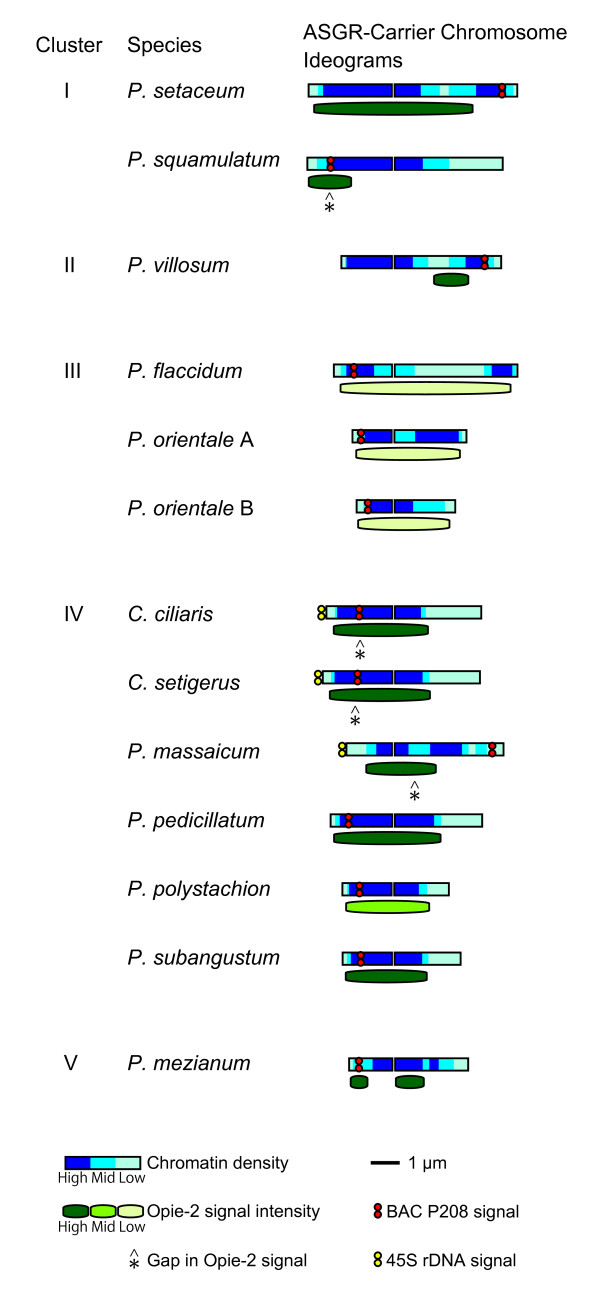
**ASGR-carrier chromosome ideograms for apomictic *Pennisetum *and *Cenchrus *species clustered according to the phylogenetic analysis**. In ideograms, dark, medium and light blue indicate chromatin condensation pattern (regions of high, middle and low condensation, respectively). Red and yellow circles indicate BAC P208 and 25S rDNA, respectively. Opie-2 distribution, as determined by P602 signal, is indicated as bars below each ideogram and intensity of shading represents the approximate intensity of P602 signal. Asterisks indicate the position of discontinuous P602 signal. Bar corresponds to 1 μm. The data of *P. squamulatum *and *C. ciliaris *are from Akiyama et al. (2004) and Akiyama et al. (2005), respectively.

The results of FISH with ASGR-linked BACs are summarized in Table 2 and Figs. 4 and 5. No sexual species showed discrete signals from hybridization of the ASGR-linked BACs P001, P109 or P208. BAC P208 showed weak signal on the centromeres of not only aposporous, but also sexual species. In aposporous species, the ASGR-linked BACs were detected as strong signals on a single chromosome (Figure 4a, c-j) with one exception (Figure 4b). In *P. orientale *(PS12), a 54-chromosome accession, two ASGR-carrier chromosomes were observed (Figure 4b). The BACs sometimes showed strong and spatially distinct signals within the ASGR indicating duplicated loci or repetitive sequences.

### Localization of 25S rDNA on the ASGR-carrier chromosome

The rDNA probe was used as a cytological marker to test whether the ASGR in species other than *C. ciliaris *[34,37] was associated with a 25S rDNA locus. Only two other species, *C. setigerus *(PS16) and *P. massaicum *(PS680) showed rDNA signals on the ASGR-carrier chromosome. The ASGR-carrier chromosome in *C. setigerus *was indistinguishable from *C. ciliaris *in the position of the rDNA locus, i.e., terminal on the short arm of the ASGR-carrier chromosome (Figure 4l), in addition to other characters (Figure 5). In *P. massaicum*, a rDNA locus was distally located on the short arm of the ASGR-carrier chromosome whereas the ASGR was terminal on the long arm (Figure 4k).

### Characteristics of the ASGR-carrier chromosome in *Pennisetum *species

FISH experiments revealed that morphologies of the mitotic ASGR-carrier chromosomes among the species were different; therefore, image analysis was carried out to quantify the differences (Table 2, Figure 5). Threshold values of gray and black levels in the ideograms were assigned to display differences in chromatin density between chromosome regions. The lengths of ASGR-carrier chromosomes ranged from 3.37 μm in *P. orientale *(PS12) to 7.20 μm in *P. setaceum *(PS25). The ASGR position was estimated based on the mid-point of the P208 signal and was always shown to be in or near a moderately to highly condensed region, again with the exception of *P. massaicum*.

**Figure 6:**
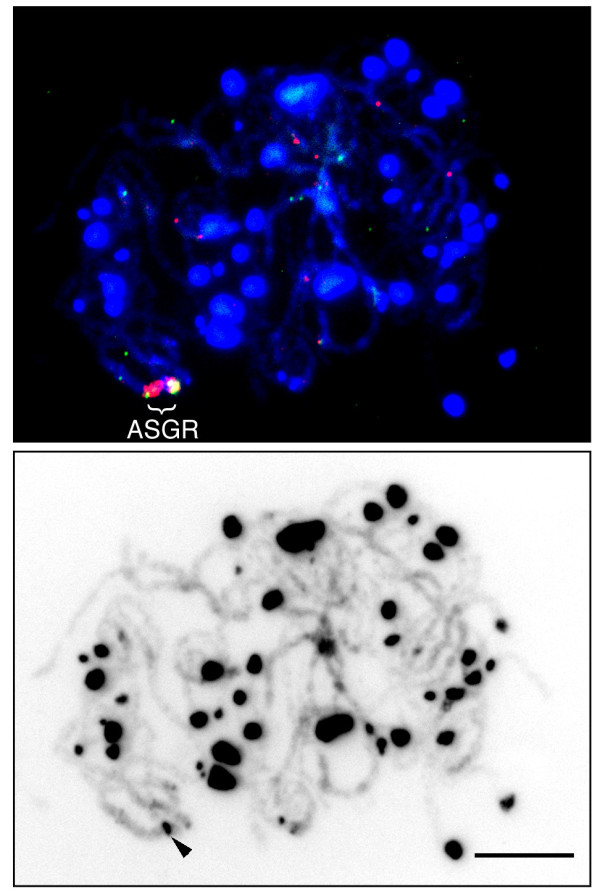
**Physical mapping of BAC clones on pachytene chromosomes of *P. orientale *(PS12)**. Upper image: Color-merged images of DAPI and FISH signals. Red and green signals are P001 and P208, respectively. Lower image: inverted DAPI image. Arrow indicates knob in the ASGR. Bar corresponds to 10 μm.

Lengths and arm ratios of the two ASGR-carrier chromosomes in PS12 were compared to each other by a paired t-test, which showed a significant difference for chromosome length (t = 3.16 P < 0.01) but not for arm ratio (t = 0.39, P = 0.70). The DNA distribution on the two ASGR-carrier chromosomes of PS12 showed different patterns as measured by DAPI staining intensity (Figure 5). The ASGR-carrier chromosome PS12a had a highly condensed heterochromatic region on the long arm that was confined to the pericentromeric area of PS12b. The ASGR itself was located on the distal end of the short arm in both ASGR-carrier chromosomes. Based on mitotic chromosome characteristics, the two ASGR-carrier chromosomes in PS12 were heteromorphic and suspected to be homeologous rather than homologous chromosomes. However, physical mapping of paired chromosomes at the pachytene stage of meiosis using BACs P001 and P208 showed that the two ASGR-carrier chromosomes formed bivalents with one another as would be expected of homologs (Figure 6). A heterochromatic knob was observed in the ASGR.

Morphology of the ASGR-carrier chromosome in *C. setigerus *was similar to that of *C. ciliaris *(Figure 5) (data from [37]) and a t-test showed no significant difference in chromosome length (t = 0.18 P = 0.85), arm ratio (t = 0.87, P = 0.38), or signal position of P208 (t = 1.08, P = 0.28). The signal position of C101, an ortholog of P208 was used in *C. ciliaris*.

**Figure 7:**
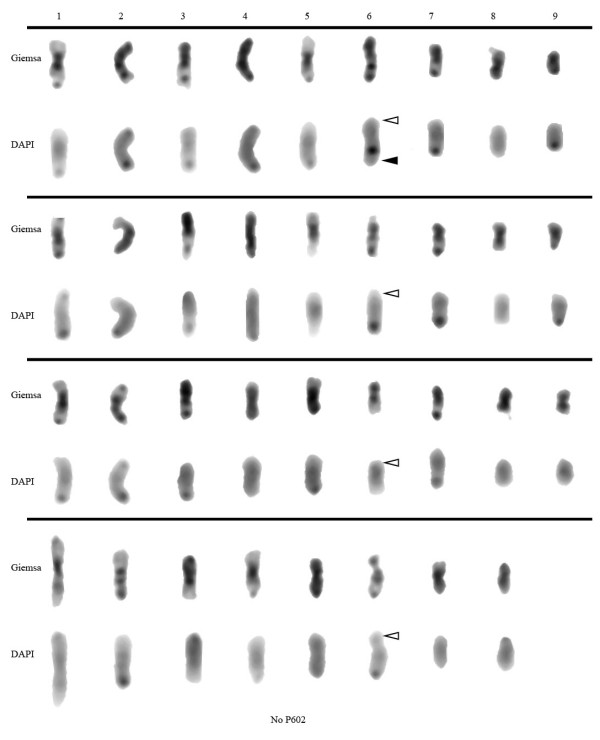
**Ordered chromosomes based on Giemsa- and DAPI-stained chromosomes of *P. massaicum *(PS680)**. These chromosomes are from the spreads of Figure 1j and k and were sorted according to their lengths. The bottom two rows of chromosomes are the eight that did not hybridize with BAC P602. Black and white arrow heads indicate ASGR and rDNA, respectively.

The ASGR-carrier chromosome in *P. massaicum *(PS680) showed unique morphology among the species with a highly condensed region in the middle of the long arm (Figure 5). PS953, another *P. massaicum *accession, showed the same ASGR-carrier chromosome characteristics as PS680. The morphology of the ASGR-carrier chromosome was also sufficiently unique within this species such that it sometimes could be distinguished under phase contrast without Giemsa staining or FISH (Figure 4j, Figure 7). Comparison of the Giemsa-stained chromosomes having rDNA indicated that morphology was different among them (Figure 7).

### Distribution of Opie-2 like retrotransposons

BAC P602 contains an Opie-2-like retrotransposon abundant only in the ASGR of *P. squamulatum*, but occurring throughout the genome of *C. ciliaris *[37]. The distribution of this repetitive sequence was clearly different in two species that often are grouped as one, *P. mezianum *(PS9) and *P. massaicum *(PS680) [26]. In *P. mezianum *with 32 chromosomes, only the ASGR-carrier chromosome showed signal from hybridization of P602 (Figure 4m), whereas in *P. massaicum*, 27 out of 35 chromosomes, including the ASGR-carrier chromosome, showed intense signal with this BAC (Figure 4k). This retrotransposon family is present at varying copy numbers in different species within each cluster, regardless of mode of reproduction (Table 2). For example, in clade V it is abundant in *P. nervosum *but barely detectable in *P. ramosum*, both sexual species. Mainly two patterns emerged in the apomicts, repeats detectable flanking the ASGR plus either distributed throughout the genome (buffelgrass pattern) or confined to the ASGR-carrier chromosome (*P. squamulatum *pattern). The latter pattern occurred in only two other species, *P. setaceum *and *P. mezianum*. The Opie-2-like repeat was of low abundance flanking the ASGR in *P. setaceum, P. villosum*, and *P. massaicum.*

### Phylogenetic reconstruction based on reproductive and cytological features

**Figure 8:**
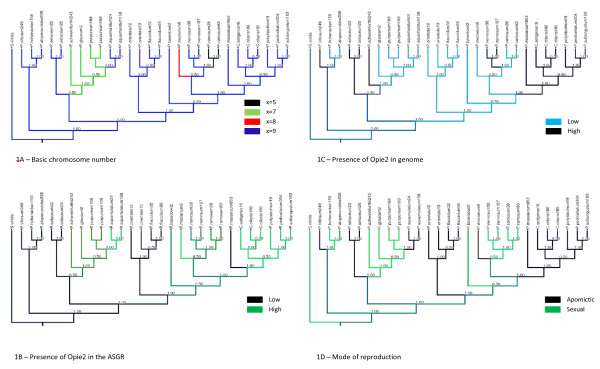
**Ancestral states for different characters**. Bayesian trees based on the *ndh*F+*trn*L-F sequence showing the ancestral state for different characters. **A**. Chromosome number, **B**. Presence of Opie-2 at ASGR, **C**. Opie-2 distribution on genome, **D**. Mode of reproduction

Reconstruction of ancestral states was done by Mesquite. Four characters viz mode of reproduction, basic chromosome number, distribution of Opie-2 on genomes, and distribution of Opie-2 on the ASGR (Table 2) were used for reconstruction of ancestral states using the tree generated by Bayesian method (Figure 8).

## Discussion

### Phylogenetic analysis

Earlier attempts to resolve phylogenetic relationships among different species in the genus *Pennisetum *or higher taxonomic levels including *Pennisetum *were based on multiple approaches such as genome size variation [39], molecular markers [40,41], and DNA sequence information from a) the internal transcribed spacer region of ribosomal DNA [24], b) the nuclear gene *knotted *[42,26], and c) the chloroplast genes *ndh*F [25,26,42-44], *rpo*C2 [45], and *rpl16*/*trn*L-F [26,27]. The present study, focused on apomixis, included twelve apomicts and eight sexual species. The sequence data obtained was carefully analyzed and resulted in a robust tree with clear groupings which can be attributed to quality sequence data. Our results show congruence with earlier studies of these genera, but also emphasize a few notable exceptions.

Chemisquy [26] recently used *trn*L-F and *ndh*F for conducting a phylogenetic analysis. A combined analysis of their sequences along with the sequences used in this study generated a tree presented in Figure 2. Combined analysis did not show any variation from the individual analysis of the two datasets. The tree from the combined analysis did show discrepancies in the placement of a few species from the present analysis as compared to the one from Chemisquy [26]. These discrepancies could be due to errors in species identification or DNA sequence.

Previous studies of nuclear and chloroplast gene sequences suggested that, within the bristle clade of grasses, the genus *Cenchrus *was monophyletic and embedded in a polyphyletic *Pennisetum *[42]. Polyphyly in *Cenchrus *was supported by additional chloroplast sequence data and taxon sampling [27]. Our data support the placement of *Cenchrus *within *Pennisetum*, but are incongruent with the result of Donadio [27] and Chemisquy [26] that showed a close relationship between one accession of *P. purpureum*, *P. setaceum*, and *Cenchrus spp*. In the ITS-based phylogeny of Martel [24], *P. setaceum *and *P. villosum *grouped with species from section Brevivalvula in a clade that included *Cenchrus ciliaris*; therefore, their placement of *P. setaceum*, but not *P. villosum*, was more consistent with Donadio [27]. According to Chemisquy [26], *P. villosum *grouped away from *Cenchrus *species and Brevivalvula section. In contrast, results from the present study place *P. setaceum *as a member of a large clade that included the cultivated species *P. glaucum *and excluded species from section Brevivalvula while *P. villosum *grouped with *P. hohenackeri *and *P. alopecuroides*. Doust and Kellogg [23] also found *P. setaceum *to be closely related to *P. glaucum*. Section Brevivalvula is considered more homogeneous compared with other sections in the genus *Pennisetum *[8] and morphologically is well differentiated from the other sections [46]. The non-concordance between relationships inferred from ITS and cpDNA data sets could be due to a number of factors. First, the ITS is part of a genomic region known to be affected by the process of concerted evolution [47]. As with incomplete lineage sorting, concerted evolution can lead to the phylogenetic association of lineages that did not share a most recent common ancestor. In contrast to the ITS, *ndh*F is a chloroplast gene and thus likely not susceptible to the process of concerted evolution, although nuclear capture of chloroplast DNA is possible [48] but untested in this case.

The placement of *P. setaceum *in the *P. glaucum *clade is supported by the current and prior [23] chloroplast phylogeny and crossability studies [49]. This clade also includes *P. purpureum*, *P. squamulatum*, and *P. schweinfurthii*. *P. squamulatum*, recently proposed by Akiyama et al. [50] to be a member of the secondary gene pool (i.e., the group of biological species that will cross with the crop species [51]), has strong support from *ndh*F and cytological [50] data for a close relationship with *P. glaucum *(the primary gene pool) and *P. purpureum *(secondary gene pool). Since revision of the basic chromosome number of *P. squamulatum *from x = 9 to x = 7 [50], all species in clade I, except for *P. setaceum*, have a basic chromosome number of x = 7 and all (including *P. setaceum*) can be crossed with *P. glaucum *[52-55]. The accessions of *P. purpureum *we investigated were strongly supported as sister to *P. glaucum*, confirming placement of this species in the secondary gene pool of *Pennisetum *[56]. Natural hybrids have been reported between *P. glaucum *and *P. purpureum*, and one genome of *P. purpureum *has been suggested to be homologous with pearl millet [57].

Based upon the *ndh*F data, the apomictic pentaploid *P. villosum *is most closely related to the sexual diploids, *P. hohenackeri *and *P. alopecuroides*. *P. hohenackeri *and *P. alopecuroides *also grouped together in an ITS-based phylogeny [24], while *P. alopecuroides *grouped with *P. villosum *based on EST-microsatellites [41] and the *knotted-1 *gene sequence [42], further supporting a close phylogenetic relationship between all three species. In contrast, *P. alopecuroides *was more closely related to *P. glaucum *than *P. villosum *based on the chloroplast sequence data of Donadio [27]. Significantly, meiotic chromosome configurations in pentaploid *P. villosum *are also consistent with its derivation through hybridization [57], and incongruence between studies could result from multiple hybrid origins or ploidy level differences among accessions.

A third group in the dendrogram consisted of two apomictic species, *P. orientale *and *P. flaccidum*. *P. orientale *and *P. flaccidum *were also closely related based upon EST- microsatellite data, even though the *P. orientale *cytotype examined was 2n = 2x = 18 and likely sexual [41], as opposed to the 6x apomictic cytotypes that we studied. The position of *P. basedowii *is uncertain. While Bayesian analysis places *P. basedowii *basal to subgroups IV and V, MP analysis shows it as sister to subgroups III, and IV/V. No previous phylogenetic studies have incorporated *P. basedowii *except for Chemisquy [26] where it grouped with *P. glaucocladum*, a species not included in the present analysis, and to which *P. flaccidum *was basal. Neither does our combined analysis (Figure 2) put the accession used by Chemisquy [26] with the one used in present study. In the combined analysis, the position of the *P. basedowii *accession used in this present study is consistent with that of our Bayesian analysis.

Clade IV included 1) all three *Pennisetum *species from section Brevivalvula (*P. polystachion*, *P. pedicillatum*, *P. subangustum*), 2) *Pennisetum *species from outside this section, *P. massaicum*, and 3) the *Cenchrus *species. The close relationship between *P. polystachion *and *P. pedicillatum *of section Brevivalvula was also indicated by ITS and SSR data [24,41]. Furthermore, intermediate morphotypes and shared chloroplast haplotypes suggest considerable gene flow between species of this section [58]. All species in clade IV contain apomictic cytotypes and have basic chromosome numbers of x = 9, except for *P. massaicum *where the basic chromosome number is unclear. *P. massaicum*, with 35 chromosomes, 8 of which do not hybridize to the Opie-2-like repeat, may be an aneuploid (4x-1) as the number of rDNA loci suggests that it is tetraploid or it may have been produced by interspecific hybridization between species with basic chromosome numbers of x = 8 (low Opie-2 abundance, e.g., *P. mezianum*) and x = 9 (high Opie-2 abundance; e.g. *Cenchrus*). Another x = 8 species, *P. montanum*, falls outside of the *Cenchrus *clade [27], but was not included in our study. All species in clade IV contain apomictic cytotypes, predicting some degree of asexual reproduction in all lineages. Hybridization between the largely asexual apomicts is possible through rare fertilization of reduced or unreduced eggs [59]. One accession of *P. orientale *used in the present study uniquely showed two ASGR-carrier chromosomes, each with a different morphology but able to pair at meiosis, and may have resulted from fertilization of an unreduced egg with reduced pollen, both carrying an ASGR. In the case of the derivation of the apomictic *P. massaicum*, such hybridization would have necessarily involved at least one parental apomictic lineage that contributed the ASGR-carrier chromosome. Pollen fertility in *Pennisetum *apomictic lineages is required for pseudogamous apomixis, in which the endosperm develops only after fertilization of the central cell. Normal male meiosis, giving rise to reduced pollen is typical, but irregular segregation of genomes through aberrant meioses also occurs. Either interspecific or intergeneric hybridization may have precipitated speciation of *P. massaicum*. Genomic *in situ *hybridization (GISH) studies could provide valuable insights regarding the putative hybrid origin of this species.

The fifth cluster contains sexual *P. ramosum, P. nervosum *and apomictic *P. mezianum *with divergent basic chromosome numbers (x = 5, x = 8 and x = 9, respectively). Apomictic *P. mezianum *did show deviation in its grouping among MP and Bayesian trees. The two sexual species in this group differ in their distribution of Opie-2-like repeats, abundant throughout the genome of *P. nervosum*, but barely detectable by FISH in *P. ramosum*. Even though *P. ramosum *has the smallest number of chromosomes of any *Pennisetum *species (2n = 2x = 10), it has one of the highest DNA contents estimated per haploid genome (2.02 pg [39]) and the highest per chromosome (0.4 pg). Genome expansion through repetitive DNA amplification is the most likely explanation, although our results indicate that Opie-2-like repeats probably played a minor role. These two species were positioned in separate clades based on the analysis of Donadio [27]. Analysis based on *trn*L-F and *ndh*F did put *P. mezianum *with *P. ramosum *but away from *P. nervosum *[26].

### Evolution of the ASGR-carrier Chromosome

Ancestral analysis shows x = 9 as the ancestral condition and the other three basic chromosome numbers (x = 5, x = 7, and x = 8) as derived states which originated independently of each other (Additional File 1 Figure S1A), which is in congruence with earlier studies [26,27]. Ancestral analysis based on reproductive behavior (Additional File 1 Figure S1D) did not resolve sexuality or apomixis as being plesiomorphic. From the sequence information generated from the ASGR and the fact that the low copy BACs are shared by all the apomictic species, it is more likely that apomixis is the result of a single event as suggested earlier [18] which spread to other species through hybridization.

The three low-copy ASGR-linked BACs used in the present study produced a discrete signal only in aposporous species confirming that these BAC sequences have conserved homology in all aposporous species of the genus *Pennisetum *and the closely related genus, *Cenchrus*. By definition, therefore, all aposporous *Pennisetum *species/cytotypes possess an ASGR, while sexual species/cytotypes of the genus lack an ASGR. These results are consistent with our previous report for *C. ciliaris*, where an ASGR, existing as a heterochromatic, largely hemizygous chromosomal region on a heteromorphic chromosome, was observed in only aposporous and not in sexual cytotypes [37]. Interestingly, the morphology of the ASGR-carrier chromosome and the position of the ASGR on the chromosome differ among the species, indicating that the ASGR-carrier chromosome has undergone rearrangement. Location of the ASGR in a telomeric and condensed region of the chromosome occurs in all clades containing apomictic cytotypes and may thus be the ancestral state. In all *Pennisetum *species, the ASGR is located near the telomere of the chromosome, while it is interstitial in *Cenchrus *species and inverted relative to *P. squamulatum *[34,35]. Given that the intercalary position and linkage with rDNA on the same chromosome arm are unique characters in *Cenchrus*, this is more likely to be a derived state. Additional data concerning the orientation of the ASGR, as ascertained using FISH on the pachytene chromosomes of all species are needed to test this hypothesis.

As explained earlier, an 800 bp region was amplified from the ASGR and analyzed to further understand the evolution of ASGR. This region was previously known to be duplicated at the ASGR in *P. squamulatum *and *C. ciliaris *[38]. The present investigation could detect two copies in some species but not all the species investigated. Additional sequencing from the locus could help to discover whether the locus is also duplicated in these species. This region of ASGR duplication is recent and happened before the ASGR was transferred between *Pennisetum *and *Cenchrus *as one of the copies from *Cenchrus *shows high similarity with *Pennisetum*. Among the species where duplication could not be detected are the three species in section Brevivalvula, which also group together based on the sequence from the ASGR suggesting that they may contain the more ancient form of the ASGR. Overall, variation assessed in this region of the ASGR is very low. Although it is possible that there is an inconstant rate of evolution between different regions of the ASGR, the level of variation detected suggests a recent origin of the ASGR. Because of the low level of variation, the tree obtained is of very low resolution (Figure 3). However, the tree could still discriminate section Brevivalvula from other species in the *Pennisetum*-*Cenchrus *complex.

Opie-2-like sequences were found to be abundant in *P. squamulatum *only at the ASGR [36]. In contrast, these sequences were associated with the centromeric regions of all chromosomes in *C. ciliaris *[37]. In both species, a repeat-poor portion of the ASGR is flanked by Opie-2-rich regions [18]. Since this repeat now has been detected as part of the ASGR in seven out of 12 aposporous species (exceptions include *P. setaceum *and *P. villosum *of clades I and II, *P. orientale *and *P. flaccidum *of clade III and *P. massaicum *of clade IV), we speculate that the association of this repeat with the ASGR was derived by either 1) translocation of the repeat-poor portion of the ASGR into a repeat-rich region of the genome or 2) transposition and accumulation of retrotransposons in proximity to the ASGR. Ancestral stage analysis (Additional File 1 Figure S1C) did not show either of the two (low and high) patterns of Opie-2 distribution on the genome as plesiomorphic, although interestingly, low abundance of Opie-2 on the ASGR (Additional File 1 Figure S1b) might be plesiomorphic. Low abundance of this transposon repeat in the ASGR species mentioned above could also be due to transposon elimination, although genome reduction seems less likely than genome expansion for recently derived asexual taxa where recombination is suppressed [17]. Paradoxically, ancient, strictly asexual taxa such as bdelloid rotifers are devoid of retrotransposons [60], and genome expansion in strictly sexual *Pennisetum *species often has exceeded that of the apomicts surveyed [39]. Transposable elements can spread more efficiently in sexually reproducing populations, although sex also affords a mechanism for purging the genome of deleterious mutations [61].

Deciphering the complex relationship between transposable element dynamics and mode of reproduction is further complicated by events of hybridization (intra- or inter-specific) involving apomicts in diploid-polyploid-dihaploid cycles [10]. Hybridization can elicit gene expression, epigenome, and genome structural changes [62] some of which have been documented in apomictic *Boechera *[63]. One explanation for a pattern of Opie-2-like repeats as identified in *P. squamulatum *and *P. mezianum*, where repeats are clustered in the ASGR but are of low abundance in the remainder of the genome, is hybridization and introgression of the ASGR from an Opie-2-rich genome to an Opie-2- poor genome. An alternative explanation is local expansion of Opie-2-rich repeats. The unique features of the ASGR-carrier chromosome in *P. massaicum*, the species most closely related to *Cenchrus*, may reflect chromosome restructuring, perhaps as a consequence of hybridization, to distance the ASGR from either heterochromatin or an Opie-2-rich region yet retain its linkage to rDNA as in *C. ciliaris *and *C. setigerus*, albeit on the opposite chromosome arm compared with *Cenchrus *species.

The ASGR is present only in apomictic cytotypes of *Pennisetum *species that either have diploid or higher ploidy sexual cytotypes [57] or are closely related to other species with only sexual cytotypes. The coexistence of sexual cytotypes, diversity of ASGR-carrier chromosome structure, and yet phenotypic similarities in the apomixis mechanism and conservation of the ASGR argue for apomixis as a character that predates speciation thus has been subject to repeated transfer via introgressive hybridization.

## Conclusions

The present investigation provides interesting insights not only on the phylogeny of genus *Pennisetum *and *Cenchrus*, but also on the origin and evolution of the ASGR. FISH results reveal structural similarity within the ASGR across *Pennisetum *and *Cenchrus *apomictic species. This similarity is further supported by an ~800 base pair sequence generated from the ASGR. The fact that apomictic species cluster with sexual species in the chloroplast sequence-based phylogeny supports the view that apomixis probably originated once and then spread through repeated hybridization between the species. The presence of different morphologies for the ASGR-carrier chromosome in different species and variation in linkage with rDNA infers that the ASGR can be translocated within the genome and those genomes can support gross chromosomal aberrations as a consequence. Polyploidy associated with apomixis likely increases tolerance of the genome to mutation and chromosomal aberrations. It has often been speculated that apomicts cannot be sustained in nature for a long period of time due to their propensity for accumulating mutations in the absence of sexual reproduction. Low rates of sequence variation at the ASGR, therefore, suggest that the ASGR might be of recent origin.

Earlier studies from *P. squamulatum *and *C. ciliaris *have shown association of high-copy Opie-2-like sequences with the ASGR. Various species within the present investigation have an ASGR containing fewer copies of Opie-2-like sequences, from which we infer that association of the Opie-2 sequence with the ASGR is only a genome specific feature rather than a unique feature associated with apomixis. Ancestral state analysis suggests that the ASGR might have originated in a genome with low abundance of Opie-2 and was transferred to a high Opie-2-copy genome while independent duplication of low-copy regions within the ASGR also occurred. *P. massaicum*, which groups with *Cenchrus spp*. and section Brevivalvula species, is a species where genomes with low Opie-2 at the ASGR and high abundance of Opie-2 across the chromosome complement coexist, and most likely were merged by hybridization. Section Brevivalvula shows variation in distribution of Opie-2 on the ASGR and throughout the genome with *P. polystachion *displaying lower Opie-2 signal than the other two species examined. These observations warrant investigation into other species from the section. Additional sequence data from the ASGR also could resolve the extent of gene duplication in this region.

In conclusion, the present investigations have provided new insights into structure of the ASGR and its evolution based on experimental evidence. Further studies in this direction can lead to information which can help in deciphering the intrigues of apomixis.

## Methods

### Plant materials

Plant materials and their origins are listed in Table 1. All species were grown in a greenhouse on the Tifton Campus of the University of Georgia. For pachytene chromosome preparation, immature panicles were collected and fixed in 3:1 ethanol:acetic acid for 2 days at room temperature. The fixed material was stored at 4°C and used within one year. For mitotic chromosome preparation, root tips of all species were collected and pretreated for 3 h by soaking in a saturated solution of α-bromonaphthalene on ice before fixation in 3:1 ethanol:acetic acid.

### Cytological analysis

Fluorescence in situ hybridization was carried out according to Akiyama [36]. Enzymatic maceration of root tips and anthers for chromosome spreads and denaturation for probe hybridization were conducted using the indicated times for each species (Table 2). ASGR-linked bacterial artificial chromosome (BAC) clones (P001 - containing SCAR A14M; P109 - SCAR Q8M; P208 - SCAR UGT197) have been described previously [35,38,64]. A BAC (P602) containing SCAR marker X18 and a large amount of repetitive DNA was isolated by PCR screening of pooled BAC DNAs [36]. The BAC clones were labeled with Biotin-16-dUTP (Roche, Indianapolis, IN) or digoxigenin-11-dUTP, alkali-stable (Roche) by nick translation. Ribosomal loci were detected using a 25S rDNA probe from rice [65] cloned in pCR 2.1-TOPO (Invitrogen, Carlsbad, CA). The pCR 2.1-TOPO insert was labeled using PCR amplification with M13 primers. Denatured chromosomes were incubated with 10 μl denatured hybridization mixture consisting of approximately 5 ng/μl biotin- or digoxigenin-labeled probe, 5% dextran sulfate, and 50% formamide in 2X SSC in a humidified chamber at 37°C overnight. After hybridization, the digoxigenin-labeled probes were detected with fluorescein using a fluorescent antibody enhancer set (Roche). Biotin-labeled probes were detected with Texas-red streptavidin (Vector Laboratories, Burlingame, CA) and biotinylated anti-streptavidin (Vector Laboratories) for a second layer of Texas-red streptavidin. After detection, the slides were mounted in Vectashield (Vector Laboratories) containing 1.5 μg/ml DAPI and observed under a fluorescence microscope, Olympus BX50. Images of chromosomes were captured by a Sensys CCD camera (Sensys Photometrics, Tucson, AZ) and Image Pro ver 4.1 software (Media Cybernetics, Silver Spring, MD). Image analysis was performed with Object-Image 2.08 (http://simon.bio.uva.nl/object-image.html) and modified CHIAS3 [66]. The statistical analysis was carried out by Microsoft Excel 98 (Microsoft, Redmond, WA).

### Amplification and sequencing of chloroplast NADH dehydrogenase, *ndh*F, *trn*L-F and *ASGR BBM-like *genes

Total DNA was extracted from fresh leaves using the procedure of Tai and Tanksley [67] or following the protocol from the DNAeasy Plant Mini Kit (Qiagen, Valencia, CA). All PCR amplifications were carried out in a GeneAmp^® ^PCR system 9700 thermal cycler (Applied Biosystems, Carlsbad, CA). The 3' end of the *ndh*F gene was amplified using the primers 972 and 2110R [29]. Amplification was carried out in 50 μl reactions containing 1X reaction buffer supplied by the manufacturer plus 2.5 μM MgCl_2_, 0.2 mM dNTPs, 1.5 U *Taq *polymerase, and 0.2 μM of each primer. Cycling conditions were denaturation at 94°C for 5 min followed by 35 cycles of 94°C for 1 min, 55°C for 1 min, 72°C for 1.5 min, and a final extension for 7 min at 72°C. The amplified products were purified using the Qiaquick PCR purification kit (Qiagen, Valencia, CA). The purified products were sequenced using primers 972, 1603, 1603R and 2110R. In a few species, primers 1318, 1318R, 1955 and 1955R were used to complete the sequencing of amplified fragments. Sequencing was carried out on a Beckman CEQ8000 (Beckman Coulter, Fullerton, CA) according to the manufacturer's instructions. The *trn*L-F region was amplified using the primers *trn*L-F_c and *trn*L-F_f as per [68]. The *ASGR-BBM-like *region was amplified using the primers p779/p780. These primers amplify only apomicts from segregating F_1 _populations of both *Pennisetum *and *Cenchrus *(unpublished results) and showed amplification only in apomictic and not the sexual species in the present study. Amplification was carried out in 50 μl reactions containing 50-75 ng template DNA, 1X iProof GC buffer, 200 μm each dNTP, 0.5 μM each of *trn*L-F or *ASGR-BBM-like *primers and 0.02 U/μl iProof DNA polymerase (Bio-Rad Laboratories, Hercules, CA). The cycling conditions were one cycle of 98°C for 30 s followed by 30 cycles of 98°C for 8 s, 60°C (*trn*L-F)/52°C (*ASGR-BBM like*) for 20 s, 72°C for 25 s with a final 72°C extension for 10 min. The amplified products were ligated with the pCR^®^-BluntII-TOPO^® ^vector (Invitrogen) and transformed into NEB *E. coli *DH5α following the manufacturer's instructions (New England BioLabs, Ipswich, MA). For each *trn*L-F DNA template 3 clones were fully sequenced. Sequencing was performed by the Georgia Genomics Facility, (Athens, GA) using M13 Forward and Reverse primers. For each *ASGR-BBM-like *DNA template 5 clones were sequenced using M13 Forward primer.

### Phylogenetic Analysis

The nucleotide sequences generated were aligned by Clustal X ver 1.81 [69]. The aligned sequences were translated and compared with protein information for *ndh*F available from the whole chloroplast genome sequence in *Nicotiana tabacum *(Accn. No. Z00044 S54304). Sequence chromatograms were cross-checked at positions where mutations were predicted. The corrected alignment was used for phylogenetic analysis. The analysis was done by PAUP 4.0 beta 10 for windows [70] and Mr. Bayes 3.1.2 [71]. Mr. Model test V2 [72] was used to decide the best-fit model for parsimony and Bayesian analysis. Parsimony analysis was done by PAUP with 100 replicates being used for bootstrap analysis. For Bayesian analysis, Mr. Bayes was used and the analysis continued until standard deviation of split frequencies became less than 0.01. For analysis of 29 taxa analyzed in this study, analysis was run for 400,000 generations while for the combined analysis of sequences with sequences from present investigation and Chemisquy et al. [26], analysis was run for 3,200,000 generations to achieve the desired standard deviation of split frequencies. All trees were viewed by the program Tree View ver 1.6.1 [73]. Mesquite ver 2.74 [74] was used to alter the tree or to do phylogenetic reconstruction based on a character to identify the ancestral state for that character.

## Authors' contributions

SG produced sequence for the *ndh*F chloroplast gene, produced and analyzed all the evolutionary data and wrote those corresponding parts of the paper. YA produced and did image analysis of the chromosomal and FISH data and wrote those corresponding parts of the paper. JC produced the sequence for the *ASGR-BBM-like *and *trn*L-F chloroplast genes and participated in their analysis and writing of results. PO-A conceived of the project, provided guidance, secured funding for the study, and coordinated manuscript preparation. WWH provided plant research material and contributed to cytological analysis. HY-A contributed with image analysis and reconfirmed the PS680 rDNA results. All authors have read and approved the manuscript.

## Authors' information

Current addresses:

YA: Livestock and Forage Research Division, Tohoku Agricultural Research Center (TARC), National Agriculture and Food Research Organization (NARO), Akahira 4, Shimokuriyagawa, Morioka, Iwate 020-0198, Japan

SG: Department of Botany, University of Delhi, Delhi 110007, India

## Supplementary Material

Additional file 1 **Name and sequence of ASGR primers tested on the species**. Click here for file
