# Supplementary material for: Evolution of the apomixis transmitting chromosome in Pennisetum
Source: BMC Evol Biol. 2011 Oct 5;11:289. doi: 10.1186/1471-2148-11-289 (PMC3198970; doi:10.1186/1471-2148-11-289)
Supplement: Additional file 1 — Name and sequence of ASGR primers tested on the species. [file 1471-2148-11-289-S1.DOC]

| Primer Name | Sequence 5’ to 3’ | Annealing Temp |
| --- | --- | --- |
| P779  P780 | TATGTCACGACAAGAATATG  TGTAACCATAACTCTCAGCT | 52°C |
| UGT197-F3  UGT197-R3 | GGATGAATAAAACGGTGTTGGGAG  AGAACAACCGCACAAGTGAGAGAA | 59°C |
| P16R-F  P16R-R | CCAAGCTGCCATATCTCCATGCTC  ATCCGGGACATGCTGTGCGATTTC | 59°C |
| RR2  RR3 | ATCACCGACTACTGGATGCCCGAGATG  CACGGACGGGCTTGAGCAGGAAATC | 59°C |
| P494  P495 | GGACCATCAGCAGGCTTACT  GAGACTAGCAGTAACTATGATCCTTAAT | 59°C |
| P207B-F  P207B-R | TTCAGAGGCTACAGTCTCCACTTG  AGGAAACAGATTTGCGATACATGA | 59°C |
| P208B-F  P208B-R | TTTCCTCTTAGAACAGAGCCAACC  AGTAACGTCTCCACGTAGCATCAG | 59°C |
| P101S-F  P101S-R | ATTGATTTTGCAGACGCGACGCTT  TTGAATTTCCTGTCCGCTCCCG | 59°C |
